# Transport of nitrite from large arteries modulates regional blood flow during stress and exercise

**DOI:** 10.3389/fcvm.2023.1146717

**Published:** 2023-06-12

**Authors:** J. C. Muskat, C. F. Babbs, C. J. Goergen, V. L. Rayz

**Affiliations:** ^1^ Weldon School of Biomedical Engineering, Purdue University, West Lafayette, IN, United States; ^2^ Mechanical Engineering, Purdue University, West Lafayette, IN, United States

**Keywords:** arteriole, autonomic stress, endothelium, nitric oxide, peripheral artery disease, vasodilation, wall shear stress

## Abstract

**Background:**

Acute cardiovascular stress increases systemic wall shear stress (WSS)–a frictional force exerted by the flow of blood on vessel walls–which raises plasma nitrite concentration due to enhanced endothelial nitric oxide synthase (eNOS) activity. Upstream eNOS inhibition modulates distal perfusion, and autonomic stress increases both the consumption and vasodilatory effects of endogenous nitrite. Plasma nitrite maintains vascular homeostasis during exercise and disruption of nitrite bioavailability can lead to intermittent claudication.

**Hypothesis:**

During acute cardiovascular stress or strenuous exercise, we hypothesize enhanced production of nitric oxide (NO) by vascular endothelial cells raises nitrite concentrations in near-wall layers of flowing blood, resulting in cumulative NO concentrations in downstream arterioles sufficient for vasodilation.

**Confirmation and implications:**

Utilizing a multiscale model of nitrite transport in bifurcating arteries, we tested the hypothesis for femoral artery flow under resting and exercised states of cardiovascular stress. Results indicate intravascular transport of nitrite from upstream endothelium could result in vasodilator-active levels of nitrite in downstream resistance vessels. The hypothesis could be confirmed utilizing artery-on-a-chip technology to measure NO production rates directly and help validate numerical model predictions. Further characterization of this mechanism may improve our understanding of symptomatic peripheral artery occlusive disease and exercise physiology.

## Introduction

Exercise and changes in emotional state (i.e., a fight-or-flight response) are associated with increases in regional blood flow via reduction of downstream vascular resistance (1). As local increases in wall shear stress (WSS) upregulate endothelial nitric oxide synthase (eNOS) expression, acute cardiovascular stress stimulates the release of nitric oxide (NO) into luminal and abluminal regions (2) where it is metabolized to nitrite (NO_2_^−^) or nitrate (NO_3_^−^) (3). Furthermore, activation of inducible NO synthase (iNOS) due to oxidative stress associated with maximal arousal substantially elevates plasma nitrite/nitrate (NO_x_) by up to 20 µM during high-intensity, dynamic exercise (4, 5). Following evidence from Lauer et al. (6) that “the NO:oxyHb reaction [and subsequent nitrate formation] plays only a minor role for the inactivation of NO *in vivo*,” we expect these changes in NO_x_ represented increases in plasma nitrite–a key regulator of vascular function (7–9).

Research by Kelm and colleagues established that nitrite, rather than nitrate, reflects regional eNOS activity in mammals (6). As nitrite infusion at resting concentrations (0.3–0.5 µM) (10) are vasodilator-inactive (6), this distinction led many groups to consider flowing blood as a sink from which endothelial-derived NO is eventually inactivated. However, Hon et al. demonstrated autonomic stress increases both consumption and vasodilatory effect of endogenous bloodstream nitrite (11), preventing endothelial dysfunction via increased NO bioavailability (12). Furthermore, evidence of conduit artery NO improving blood flow in the microvasculature of canines (13) and rodents (14) suggests that advection (transfer by fluid flow) of nitrogen oxides to downstream resistance vessels can promote vasodilation.

Gladwin and others have since demonstrated that nitrite acts as a major bioavailable pool of NO, nitrite reductase activity is augmented during hypoxia, and nitrite acts as a relatively potent and fast vasodilator at near-physiological concentrations (1.0–2.0 µM) (15–20). Thus, these previous reports suggest that bloodstream nitrite participates in maintaining intravascular NO homeostasis following acute cardiovascular stress. In this article, we present a hypothesis that considers the possibility that accumulation of nitrite in the cell-free plasma layer raises near-wall nitrite concentrations to levels sufficient for vasodilation as blood flows from large arteries to small arterioles during cardiovascular stress and exercise.

## Hypothesis

We hypothesize that during acute cardiovascular stress or strenuous exercise, enhanced production of NO by vascular endothelial cells raises nitrite concentrations in near-wall layers of flowing blood resulting in cumulative NO concentrations sufficient for vasodilation.

### Explanation of hypothesis

**Figure 1:**
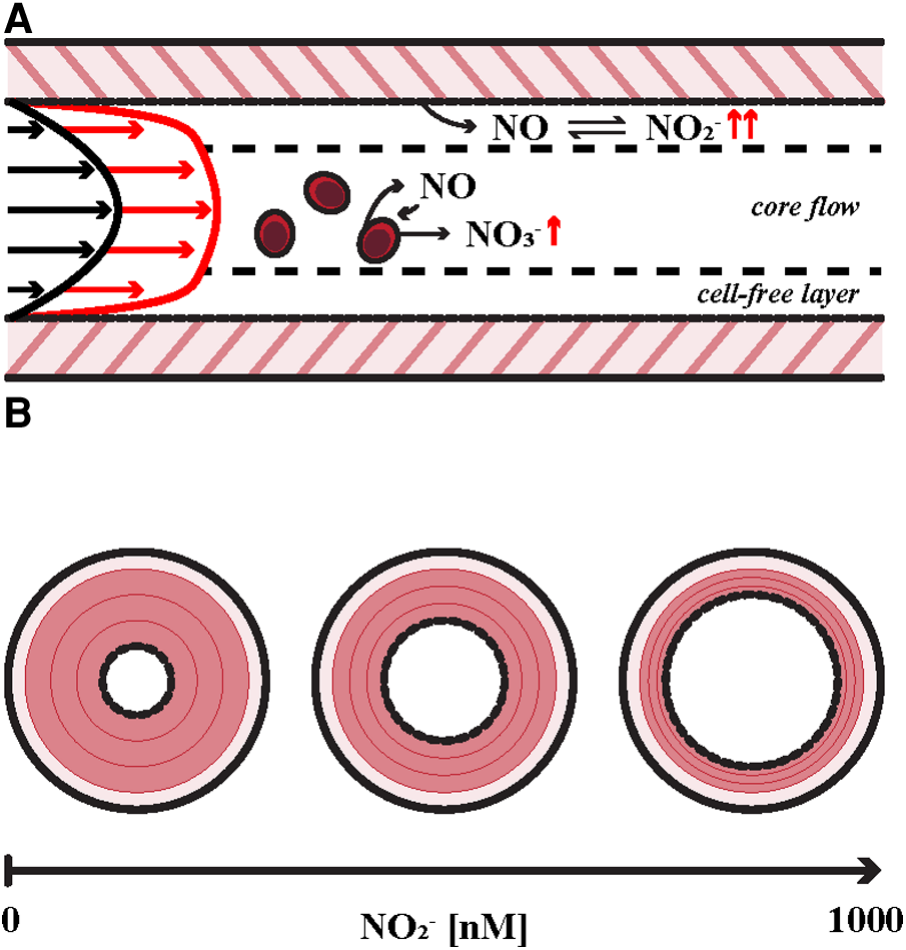
Key aspects of intravascular metabolism of endogenous nitric oxide (NO). (**A**) Hyperemia is the result of a reduction in peripheral vascular resistance that is induced by increased wall shear stress (WSS) in larger conduit arteries associated with cardiovascular arousal such as aerobic exercise as indicated by velocity profiles at rest (black arrows) and exercise (red arrows). Endothelial nitric oxide synthase (eNOS) expression is upregulated in response to local WSS augmentation, resulting in enhanced NO release into the bloodstream. NO is then converted to nitrite (NO_2_^−^) in the cell-free plasma layer and in nearby red blood cells (RBCs), which either store nitrite in equilibrium with biologically active NO or inactivate a small portion via oxidation to nitrate (NO_3_^−^). (**B**) The effect of plasma nitrite on vessel tone. Smooth muscle relaxation is illustrated in red. Vessel caliber is modulated by physiologic levels of plasma nitrite [∼500 nM (10)] in mammals. Dejam et al. identified a vasodilatory first-pass concentration of 1,000 nM in human subjects (15, 17–20).

Following the onset of acute autonomic stress or exercise, changes in arterial hemodynamics increase NO release by endothelial cells. Half of endogenous NO diffuses into the vascular smooth muscle, resulting in local vasodilation, while half diffuses into the cell-free plasma layer of flowing blood (2). Here NO, with a half-life ∼2.0 msec (21), is predominantly oxidized to nitrite, with a half-life of approximately ∼40 min (15), indicating that deactivation by cell-free or red cell oxygenated hemoglobin (oxyHb) occurs later in the NO-nitrite-nitrate lifecycle (6). Antegrade blood flow carries nitrite downstream and raises near-wall nitrite levels in arteriolar resistance vessels. Nitrite accumulation in these vessels reach vasodilator-active levels during the first pass of blood flow, promoting increased cardiac output, performance, and survival in mammals during cardiovascular arousal. Figure 1 summarizes key components of the hypothesis related to intravascular NO homeostasis.

## Support for the hypothesis

• Nitrite is a potent vasodilator at near-physiological concentrations (>1.0 µM) in mammals. It functions as an endocrine reservoir of NO, producing remote vasodilation during first-pass perfusion of the contralateral limb following nitrite infusion (15). Furthermore, there is evidence that nitrite formation at the surface of endothelial cells serves to preserve endogenous NO (6, 10), and formation of vasoactive plasma nitrosothiols induce vasodilation in distal microvasculature in felines (22). This supports the assumption that endogenous NO is preserved as nitrite upon entering the bloodstream and regenerates NO in distal vasculature.
• Lauer et al. demonstrated nitrate levels are unaffected during acute stimulation with acetylcholine while nitrite levels increase significantly (6); in a later study, the authors concluded that 70% of plasma nitrite is derived from eNOS after demonstrating NOS-inhibition is accompanied by an 80% decrease in plasma nitrite concentrations (10). Obversely, there is a large 20 μM increase in nitrite/nitrate levels during high-intensity, dynamic exercise (5). Hon et al. revealed exercise in healthy humans is associated with enhanced nitrite consumption, leading to lower arterial pressure and increased cardiac output (CO) (11). Taken together, these results highlight the importance of nitrite during cardiovascular arousal.
• It follows that nitrite infusion enhances blood flow to metabolically active tissues such as the brain or skeletal muscle. Rifkind et al. found nitrite infusion to N^G^-L-nitro-arginine methyl ester (L-NAME) inhibited rats to decrease mean arterial pressure by 96% and increase cerebral blood flow by 13% (23). Cannon et al. later demonstrated that inhalation of NO raises arterial nitrite levels by 11% and eNOS inhibition during exercise reduces local blood flow by ∼25% (24). It is thought that nitrite offers a passive system of maintaining circulatory function at rest and during cardiovascular arousal, supported by arteriovenous gradients in nitrite (25).
• In addition, there is evidence of cumulative transport of nitrogen oxides *in vivo*. Local suppression of NO production in large arterioles with diameter over 500–1,000 μm reduced NO concentration by nearly 40%; moreover, Bohlen et al. suggested as much as 60% of wall NO in small arteries was from blood transport (14). In earlier work, Bohlen identified a ∼50 nM increase in perivascular NO in venules above proximal arterioles (26). The inverse relationship between perivascular NO and vessel caliber was later confirmed with microelectrode microcirculatory mapping (27).

## Evidence against the hypothesis

• Rassaf et al. demonstrated infusion of NO into the brachial artery increased plasma nitrite by 30-fold but lacked intrinsic vasodilator action (28); however, intraarterial infusion of nitrite raised plasma levels to only 0.5 μM in their study, which is beneath the first-pass concentration of 1.0 μM identified by Dejam et al. (15).
• Nitrite reductase activity, and subsequent NO regeneration by red blood cells (RBCs), the major intravascular storage sites of nitrite (9), is maximized at 50% oxygen saturation (8); of note, distribution of oxygen tension in the pial microvasculature of rats suggested oxygen saturation remains above 90% in branches greater than 26 μm in diameter at normoxia (29). As reaction with oxyHb outcompetes molecular oxygen by an order of magnitude (6), it is unlikely that nitrite reductase activity associated with deoxygenated hemoglobin (deoxyHb) occurs in upstream resistance arteries. However, compartmentalization of Hb in RBCs reduces the rate of NO inactivation by ∼1,000-fold (15).
• Exercise, and especially footstrike during running, is associated with significant increases in cell-free Hb due to hemolysis (30, 31). Minneci et al. revealed plasma nitrite inhibits hemolysis-derived vasoconstriction and potentiates vasodilation at plasma Hb concentrations less than 25 μM (32). It is plausible that increased NO release associated with acute cardiovascular stress (4, 5) serves to reduce the mass transfer between the vessel wall and bloodstream, elevating abluminal NO concentration as proposed by Bohlen et al. (14). Their hypothesis supports the notion that plasma NO/nitrite augments vasodilation via limiting diffusion from the endothelium to flowing blood.

## Evaluation of the hypothesis

**Figure 2:**
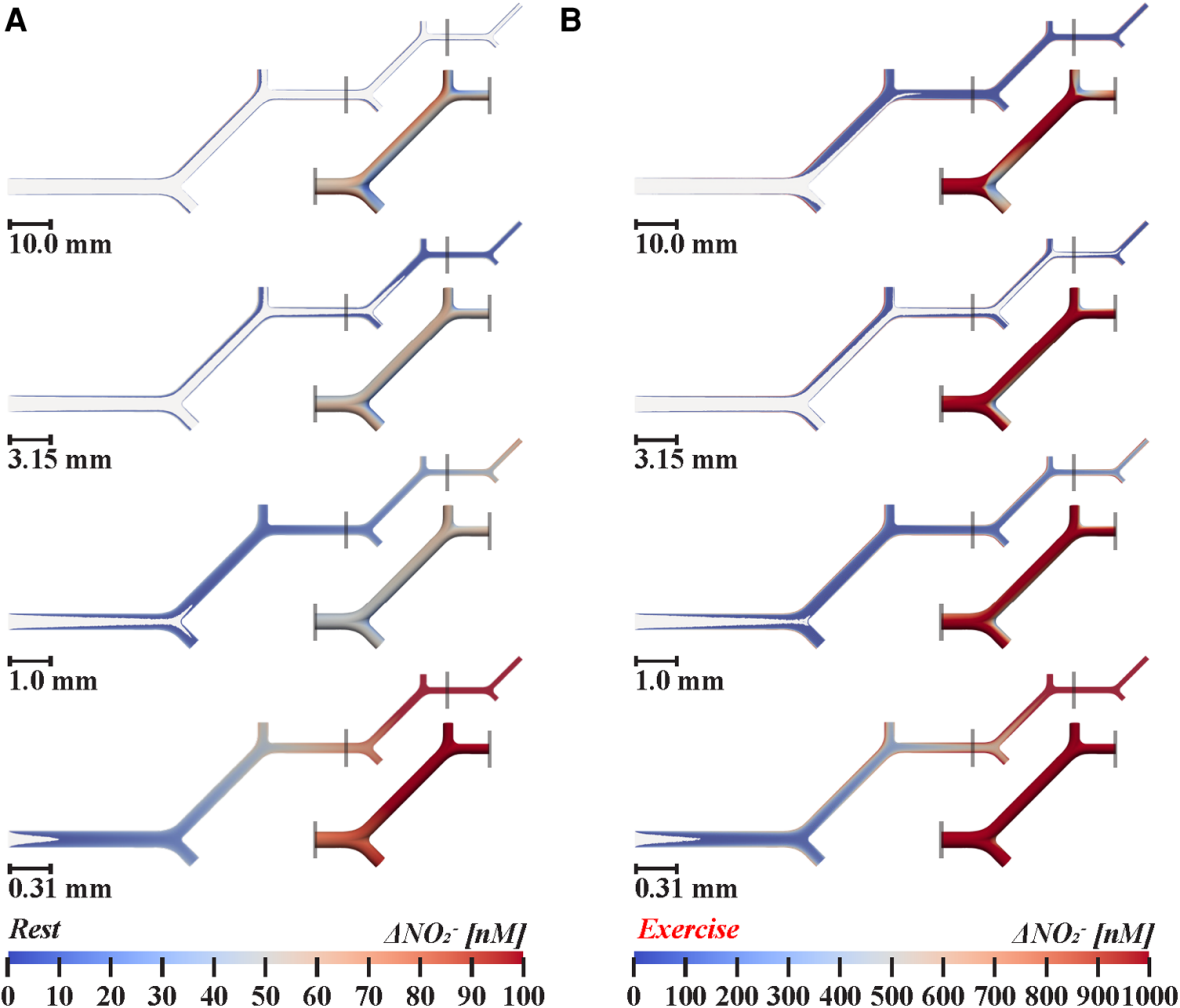
Nitrite transport in twenty-orders of bifurcating vasculature during (**A**) rest and (**B**) exercise. Two-dimensional cross-sections show luminal nitrite concentration changes above 1 nM. Insets, below and to the right of smaller branches detail how nitrite levels fluctuate at bifurcations in three-dimensions, demonstrate the impact of bifurcations on near-wall nitrite levels. 1.0× scale models simulated arteries between 1.26–4.0 mm in diameter, 0.315× between 0.40–1.26 mm, 0.099× between 0.13–0.40 mm, and 0.031× between 0.04–0.13 mm, respectively. Following *in vivo* branching patterns (33–35), the diameters of symmetrical daughter branches are 0.79 times the diameter of the parent vessel, according to Murray's law. Bifurcations facilitate local mixing of the concentration boundary layers with core flow, resulting in a 10%–38% reduction in near-wall nitrite in arteries greater than 0.20 mm in diameter. During exercise, increased velocity is associated with additional mixing in large arteries, reducing wall nitrite concentration by 20%–50% in arteries greater than 0.20 mm in diameter. In small arterioles less than 0.16 mm in diameter, the ratio of concentration boundary layer thickness to vessel diameter is increased, thus augmenting relative nitrite levels in core flow.

The following section, accompanied by Figure 2, details our multiscale model of nitrite transport in bifurcating arteries, developed to determine if intravascular transport of nitrite from upstream endothelium could result in physiologically active levels of nitrite in downstream resistance vessels.

### Method

We built a sequential multiscale model for twenty-orders of idealized bifurcating arteries (proximal inlet diameter − 4.0 mm; distal outlet diameter − 0.04 mm) following Murray's law for area ratio and a length ratio of 1.26, in agreement with *in vivo* branching patterns (33, 36). Branches were symmetrical with a bifurcation angle of 45°. The inlet velocities for rest and exercise conditions were set as 0.1 and 0.4 m/s, respectively, following *in vivo* measurement of femoral artery blood velocity at the onset of exercise (37, 38). Outflow was evenly distributed across the bifurcating branches. Due to computational limitations, we simulated six-orders of branches at a time and passed velocity information to the next smallest set of branches. Therefore, the initial model was scaled by factors of 0.315, 0.099, and 0.031 to generate a series of smaller scale models representing distal vasculature.

Computation of the generation and movement of NOx species was performed with the finite-volume solver ANSYS Fluent v18.1 (Ansys Inc., PA, United States). The heat-mass transfer analogy was used to facilitate our computational fluid dynamics (CFD) simulations (39). The heat-mass transfer analogy refers to the similarities between the linearized Fick's law of diffusion for molecular mass transfer and Fourier's law of heat conduction. Under identical flow conditions with matching of the nondimensional Schmidt number (the ratio of momentum and mass diffusivity) and Prandtl number (the ratio of momentum and heat diffusivity), the advection-diffusion solution is analogous to the mass-heat transfer solution. An unstructured tetrahedral mesh with a refined boundary layer was generated in HyperMesh 2021 (Altair Engineering Inc., MI, United States) with a target edge length of 100 μm leading to a total of 3.51 million elements. Mesh independence was verified as less than 5% deviation in wall nitrite concentration predicted on a refined mesh with a 50 μm edge length. Arterial flow was assumed to be steady and laminar, with blood modeled as an incompressible Newtonian fluid with density (ρ) of 1,060 kg/m^3^ and viscosity (µ) of 4.5 mPa·s. The diffusion coefficient of nitrite in whole blood was assumed to be 3.30 × 10^−9^ m^2^/sec (40, 41). We assumed all NO entering the bloodstream is immediately converted to nitrite with a resting and exercised rate of NO release taken as 0.00015 and 0.0068 nmol/cm^2^/sec, respectively (2, 42). Thickness of the concentration boundary layer was estimated using dimensional analysis as the square root of nitrite diffusion coefficient multiplied by vessel length divided by average velocity. We calculated mean nitrite concentration at the vessel wall, halfway along each daughter branch.

### Results

In muscular arteries greater than 0.3 mm in diameter, the Reynolds number (Re), defining the ratio of inertial and viscous forces in the flow, increased with local diameter (Re_rest_ = 1–94; Re_exerise_ = 4–377); in resistance arteries less than 0.3 mm in diameter, Re was less than 2 across both modeled cardiovascular states. Furthermore, the Peclet number (Pe), defining the ratio of diffusion to advection characteristic times, was consistently greater than 1 (Pe_rest_ = 12–1.21 × 10^5^; Pe_exercise_ = 48–4.85 × 10^5^), indicating cardiovascular nitrite transport is advection dominated (43). Pe was less than 1,000 in arterioles smaller than 0.4 mm in diameter at rest and smaller than 0.2 mm in diameter during exercise. Under resting conditions, the concentration boundary layer is estimated to be 0.02–0.03 mm thick in all branches. During exercise, this thickness decreased to 0.01 mm due to increased blood velocity.

**Figure 3:**
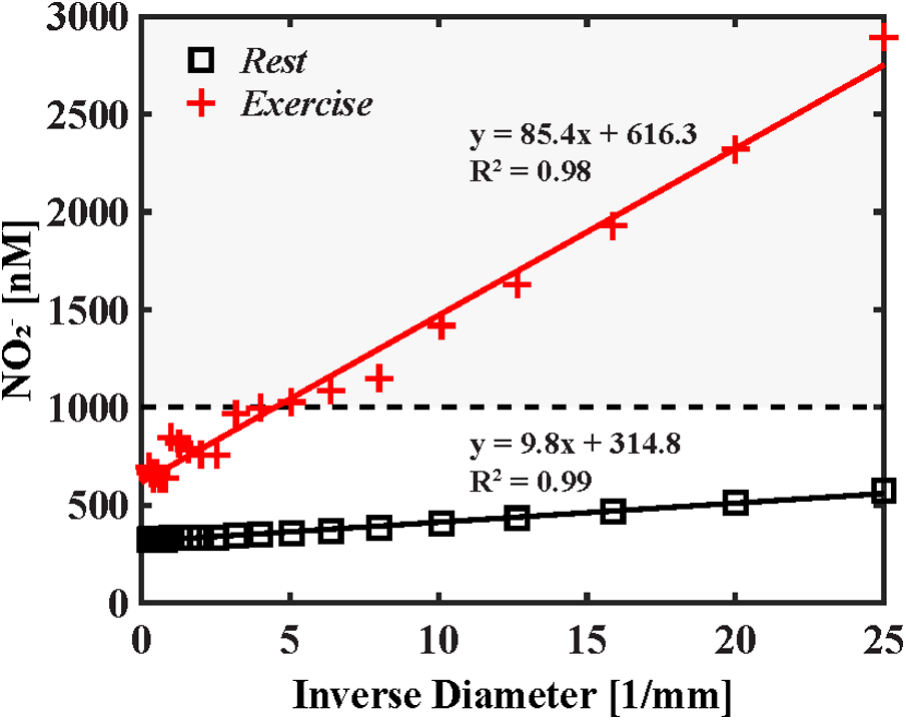
Cumulative wall nitrite concentration as a function of inverse vessel diameter. The results for rest and exercise are displayed in black squares and red cross marks, respectively. First-pass vasodilator-active concentration of 1,000 nM identified by Dejam et al. (15) is represented by the dashed line. At rest cumulative axial nitrite levels fail to reach first-pass vasodilator-active levels (represented by the horizontal dashed line); however, during exercise, near-wall concentration reaches vasoactive levels in arterioles less than 0.2 mm in diameter. Mixing associated with large arteries is reduced here, resulting in a linear increase in wall nitrite concentration in resistance arteries and arterioles.

**Figure 4:**
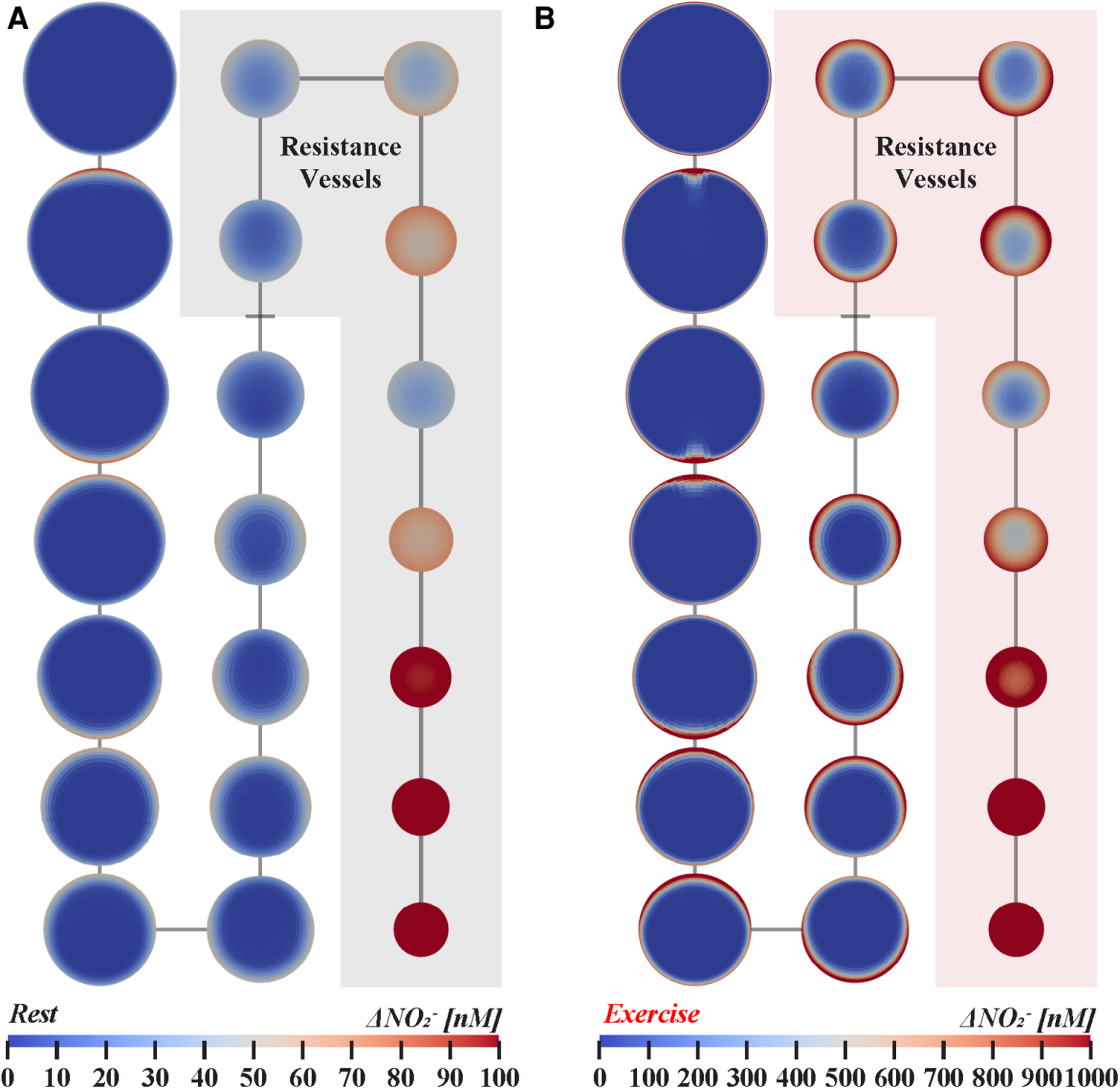
Nitrite transport in twenty-orders of bifurcating vasculature at (**A**) rest and (**B**) exercise. Cross-sections are not shown to scale for visual clarity and represent nitrite concentration halfway along each branch. Resistance vessels, 0.01–0.3 mm in diameter, are highlighted for each state of cardiovascular stress. Arteries with diameters between 1.0–4.0 mm, 0.20–0.80 mm, and 0.04–0.16 mm are shown in the left, middle, and right columns, respectively. The simulations indicate that luminal nitrite concentration can reach vasodilator-active levels during exercise.

Due to mixing with core flow, wall nitrite concentrations at the cruxes of branch points at bifurcations decrease 29%–38% at rest and by 38%–50% during exercise in arteries greater than 1.26 mm in diameter. For both simulated cardiovascular states, mixing with core flow diminishes along the arterial tree and was minimal in arterioles smaller than 0.2 mm; with no noticeable mixing in arterioles less than 0.2 mm, as shown in Figure 2A. Relative change in nitrite, defined as the difference in concentration halfway along each parent and daughter branch, indicated successive wall nitrite levels decrease down to 30 nM in arteries greater than 0.5 mm during exercise. As demonstrated in Figure 3, nitrite levels are not sufficient for vasodilation (dashed horizontal line) in all modeled vasculature scales during rest. However, due to increased rate of NO/nitrite release during exercise, simulations indicate cumulative nitrite levels do attain vasoactive levels in arterioles less than 0.2 mm in diameter (see Figure 4). Nitrite concentrations at the vessel wall are inversely related to vessel diameter in smaller arteries and arterioles (see Figure 3).

## Further testing of the hypothesis

The quantitative hypothesis developed here, in concert with preliminary CFD results, suggests further experimental studies are necessary to quantify the rate of endothelial-derived NO release at rest and during cardiovascular stress or exercise. To confirm or refute the hypothesis, it is crucial to address the orders of magnitude variation in mammalian NO production rates indicated in literature, e.g., species, culture methodology, and cell harvest location along the arterial tree modulate rate of NO production (44–46). Utilizing recent advancements in artery-on-a-chip technology (47), future studies could quantify magnitude of variation in the rate of NO release potential along the arterial tree. Compact microfluidic platforms closely resemble *in vivo* conditions and enable comparison across mammalian species in a highly replicable manner. Furthermore, these systems would allow for controlled assessment of eNOS expression in intact blood vessels exposed to resting and aroused levels of WSS.

In addition, it is plausible that bloodstream nitrite contributes to the nitrodilator-activatable intracellular NO store (NANOS) within vascular smooth muscle cells, potentiating function and contraction properties independent of local endothelial activity (48). However, how circulating nitrite stimulates release of NO from the NANOS, and the chemical nature of the NANOS, remain unknown. Again, artery-on-a-chip technology seems well suited to investigating the endogenous nitrite-NANOS lifecycle.

Future studies, despite ambiguity about the rates of NO release, could confirm the presence of additive near-wall nitrite transport (see Figure 2) through targeted disruption of NO metabolism via local eNOS inhibition in resistance arterioles (0.01–0.3 mm in diameter), as CFD results suggest nitrite accumulation is sufficient for vasodilation at this arterial scale. For example, transfer of nitrogen oxides from upstream femoral microvasculature of canines promotes vasodilation *in vivo* (13). Increased NO accumulation at these locations (as shown in Figure 3) is expected to decrease downstream vascular resistance and increase regional blood flow.

## Discussion

The present hypothesis considers NO release into the bloodstream (and subsequent equilibration with nitrite) as an evolutionary mechanism for local regulation of downstream vascular resistance during acute cardiovascular stress. Increases in heart rate and pulse pressures associated with exercise are accompanied by increases in the oscillatory shear index and magnitude of WSS (49), which is associated with a 14-fold increase in NO release (50). Under fully developed laminar flow conditions, much of the released NO/nitrite remains concentrated in near-wall fluid laminae, in which nitrite levels can accumulate to vasoactive concentrations in downstream resistance vessels during cardiovascular stress. This local synthesis and delivery minimize waste of endogenous NO. The resulting vasodilation lowers regional vascular resistance and, in turn, increases regional blood flow as well as WSS in the parent branches, creating a positive feedback effect that causes further arterial NO release.

Across a variety of species and tissues, precapillary vessels 0.01–0.3 mm in diameter account for the arteriovenous pressure drop in mammals (51). These “resistance vessels” modulate peripheral vascular resistance and regional cardiac output and, of interest, are the location where we found that nitrite accumulation first achieved vasodilator-active levels under stressed conditions (see Figure 3). These results support the hypothetical mechanism where local accumulation of bloodstream nitrite enhances perfusion of metabolically active tissues during arousal.

A confirmation of the hypothesis has implications in exercise physiology, atherosclerotic disease, and the survival benefit of a complete circle of Willis (52). Endothelium-derived, NO-mediated vasodilation is rate-sensitive with dependency on both frequency and amplitude of the shear stimulus (53–56). Of interest, the “beat” phenomenon due to cardiolocomotor synchrony in healthy runners brings about wide pressure pulse oscillations that give rise to negative arterial pressures (−20 mmHg diastolic pressure) (57). As cardiolocomotor synchrony significantly reduces run-time by 35 s on average over three miles (58) and periodic acceleration increases plasma nitrite levels over 5.7-fold (59), we speculate systemic nitrite formation is significantly elevated due to the correspondingly greater oscillatory WSS. Further, when negative arterial pressure swings occur, it is expected that monophasic antegrade flow in resistance vasculature will reverse, further stimulating the endothelium of vascular beds to release NO into the bloodstream and allowing a second pass effect, in which the same near wall fluid can receive two doses of endothelial NO, one prior to flow reversal and the other during and after flow reversal.

Peripheral artery occlusive disease due to atherosclerosis causes intermittent claudication with symptomatic pain in 5% of the population over 50 years of age (60). Upstream atherosclerotic plaques cause a pressure drop, subsequently reducing blood flow and, therefore, WSS-mediated nitrite formation and mixing with core flow (61), potentially reducing exercise tolerance in these patients. Intermittent claudication or crampy leg pain in response to walking may in part be mediated by disruption of nitrite concentration boundary layers and nitrite formation by disturbed flow distal to stenotic regions. Interestingly, Böger et al. demonstrated restoration of NO formation by L-arginine infusion improved pain-free walking distance by 230% and absolute walking distance by 155% (62). Allen et al. later hypothesized peripheral artery disease induces a reduction in plasma nitrite which is coupled to distal tissue ischemia and claudication pain following acute exercise stress; the authors demonstrated that improved “plasma nitrite flux” resulted in improved exercise performance, peak walking time, and maximal oxygen uptake (60). Taken together, these reports suggest intermittent claudication pain as an indicator of reduced nitrite bioavailability.

The circle of Willis allows blood to be redistributed throughout the brain during local ischemia; however, we and others have argued that the anatomic persistence of the circle of Willis across animal species is unlikely to be driven by natural selection of individuals with resistance to cerebrovascular disease typically occurring in elderly human populations (52, 63). Our previous work exploring the effect of anatomy and changes in frequency content of the pulse waveform (i.e., during fear and aerobic exercise) revealed a complete circle of Willis augments vertebrobasilar blood velocity acceleration 82%–134% (52). As noted above, NO-mediated vasodilation is highly rate-sensitive. Therefore, a confirmation of the present hypothesis would provide evidence that local hemodynamic changes induced by a complete circle of Willis are capable of enhancing vascular bed perfusion in the cerebellum and brainstem reticular activating system, which are critically important in survival scenarios (64).

A potential limitation of our computational models is the assumption of a rigid wall. Due to the compliant nature of large muscular arteries, radial expansion will reduce near-wall nitrite accumulation due to mixing with core flow. However, our validated reduced-order model of the systemic circulation (52) indicates femoral artery diameter amplitude (systolic minus diastolic diameter) changes less than 8.5% or less than 0.065 cm during moderate aerobic exercise. Therefore, the rigid wall assumption could be considered reasonable in the present context. The present results are representative of time-averaged steady state nitrite concentration expected to be delivered over the entire duration of exercise.

## Conclusion

We hypothesize that during acute cardiovascular stress or strenuous exercise, plasma nitrite levels accumulate in near-wall laminae to achieve vasodilator-active concentrations. Previous studies have indicated that plasma nitrite participates in maintaining vascular homeostasis during exercise, while disruption of nitrite bioavailability leads to clinical manifestations of ischemia. Herein we provided quantitative evidence for nitrite transport in twenty-orders of bifurcating vasculature under time-averaged flow conditions for the femoral artery. Nitrite accumulation in these vessels can reach vasodilator-active levels during the first pass of blood flow, which are expected to promote increased regional blood flow, cardiac output, performance, and even survival in mammals during cardiovascular stress and exercise.

## Data Availability

The raw data supporting the conclusions of this article will be made available by the authors, without undue reservation.
